# Singular Case of Recovery, after the Loss of a Considerable Portion of the Brain

**Published:** 1814-09

**Authors:** Robert Harrup

**Affiliations:** Surgeon


*For the Medical and Physical Journal.*

### *Singular Case of Recovery, after the Loss of a considerable Portion of the Brain; by* Mr. ROBERT HARRUP, Surgeon.

JAMES HAMPTON, a boy between six and seven years of age, was brought to me, on Saturday morning the 15th of May, 1813, with a wounded head. His mother gave the following account of the accident:—That yesterday afternoon a lad in play threw a stick, which, glancing from an arch, struck him on the left side of the forehead. That he was stunned for some time, and vomited and retched at intervals for several hours, which caused a great effusion of blood from the wound, as well as a quantity of *thick matter*. That with much difficulty she at last succeeded in stopping the violence of the bleeding, by binding handkerchiefs around his his head; but it still continued to come from under the clothes in drops which ran down his face. He had passed a restless night, with frequent starting and screaming.

When I saw him he was much heated, having *walked* the distance of two miles at a hurrying pace. Upon removing the handkerchiefs, a portion of the brain, as much as would have filled a large tea-spoon, lay upon that which was in immediate contact with the wound; and about half the quantity issued from the opening. His mother observed, "that is the sort of *matter* which came away last night." Being asked in what quantity, she replied, about as much as would have filled a small tea-cup. The wound was situated on the os frontis, near the sutura coronalis, some inches from the left eye-brow. The opening through the bone was circular, somewhat more than an inch in diameter. The brain here pulsating with great violence, forced out small portions of itself every instant, with a considerable quantity of bloody serum. The surrounding depression of the cranium sufficiently confirmed the assertion of the mother, that a small tea-cupful of the brain had been lost the preceding evening. Lint was applied to the opening, covering it with a slice of dry spong, which was retained by a bandage drawn moderately tight. His mother was directed to *carry* him home, and to put him to bed immediately. The antiphlogistic regimen was strictly enjoined.

Sunday, 16th.—Passed a restless night, frequently starting and screaming; pulse 112; no motion for two days past.

℞. Pulv. Jalap. ℈j. s. statim et rep. 3tia quaque horâ donec alvus respond.

Monday, 17th.—Symptoms same as yesterday. No evacuation by the bowels. Habeat quam primum Pulver. Jalap. gr. x. Calomel, gr. v. Pulver. Zingiber. gr. iii. M.

Tuesday, 18th.—Physic operated copiously three times; is much better this morning; slept well in the night; appetite good; pulse 100; wishes to get up; brought down stairs, and sat up without any uneasiness while the wound was dressed; healthy appearance around the edges of the wound; pulsation of the brain much diminished, some portion of which came away on the lint; discharge profuse; little or no complaint; dressed as before; intellect uninjured; pupils not affected, nor any other bad symptom present.

Thursday, 20th.—Continues better since last report; found him sitting up by the fire apparently in health; wound of a healthy appearance; discharge moderated; in the centre a portion of the brain of a conical shape projects, which pulsates slightly; extracted, from this part, a splinter of bone, which brought with it a portion of the brain about the size of a large pea, without the patient once complaining.

Monday, 24th.—Has continued better since last report till last night, when he waked frequently with starting and screaming; pulse 100, and somewhat full; appetite good; bowels regular; wound dressed this morning; has discharged a great quantity of well-digested pus; looks healthy; only about the size of a large pea of the brain to be seen, which pulsates more than before; the other parts of the opening filled up with healthy granulations.

Tuesday, 25th.—Found the little patient out of doors this morning, who rested well last night; bowels regular; as the wound had discharged considerably last night, dressed it again this morning; brain pulsating violently, by which a quantity of thin yellowish fluid is thrown out.

Thursday, 27th.—Still a considerable discharge from the aperture, although in a healing state; considerable pulsation.

Saturday, 29th.—Wound closing fast; discharge much diminished.

Monday, 31st.—Same as last report.

Wednesday, June 2d.—Last night fell head-foremost over a wall about five feet high, by which he was rendered sick and faint for some hours. Slept well afterwards during the night, and seems to have quite recovered this morning; wound continues to heal.

Friday, 4th.—On dressing the wound this morning, found a black hard substance on the lint, which proves to be a piece of wood, about a quarter of an inch in length, and half as wide. Two more pieces about the same size were extracted from the small opening in the centre of the wound, without the patient experiencing any pain.

Sunday, 6th.—In other respects the same as at last report.

Tuesday, 8th.—The pulsation entirely ceased.

Thursday, 10th.—Some small degree of pulsation; did not rest well last night, and had no appetite for breakfast this morning.

Sunday, 13th.—Extracted another rugged and angular piece of wood, about the size of a large pea; slight degree of pulsation.

Tuesday, 15th.—Extracted two more pieces of wood, which were firmly imbedded in the granulations.

Thursday, 17th.—Wound somewhat black in the centre, probably arising from another piece of wood approaching the surface.

Sunday, 20th.—Extracted another piece of wood from the centre of the wound, somewhat larger than any of the former.

Friday, 25th.—The granulations, which have for some time past been much higher than the surrounding skin, are now much reduced in consequence of a blow he received on the part yesterday.

From this time nothing particular occurred. The wound was firmly cicatrized on Tuesday August 17th, leaving a very considerably depression.

This boy had the measles severely the latter end of June last. He is now in perfect health. The depression in the skull is much shallower. After taking hard exercise, a pulsation is still very perceptible through the integuments of the part injured. ROBERT HARRUP.

*Chobham, August* 6, 1814.

